# Zika virus outbreak in the Pacific: Vector competence of regional vectors

**DOI:** 10.1371/journal.pntd.0006637

**Published:** 2018-07-17

**Authors:** Elodie Calvez, Laurence Mousson, Marie Vazeille, Olivia O’Connor, Van-Mai Cao-Lormeau, Françoise Mathieu-Daudé, Nicolas Pocquet, Anna-Bella Failloux, Myrielle Dupont-Rouzeyrol

**Affiliations:** 1 Institut Pasteur de Nouvelle-Calédonie, URE-Dengue et autres Arboviroses, Nouméa, New Caledonia; 2 Institut Pasteur, Arboviruses and Insect Vectors Laboratory, Paris, France; 3 Unit of Emerging Infectious Diseases, Institut Louis Malarde, French Polynesia & Aix Marseille Univ, IRD, AP-HM, SSA, VITROME, IHU-Méditérranée Infection, Marseille, France; 4 Institut de Recherche pour le Développement, UMR MIVEGEC IRD, CNRS, UM, Montpellier, France; 5 Institut Pasteur de Nouvelle-Calédonie, URE-Entomologie Médicale, Nouméa, New Caledonia; Institute for Disease Modeling, UNITED STATES

## Abstract

**Background:**

In 2013, Zika virus (ZIKV) emerged in French Polynesia and spread through the Pacific region between 2013 and 2017. Several potential *Aedes* mosquitoes may have contributed to the ZIKV transmission including *Aedes aegypti*, the main arbovirus vector in the region, and *Aedes polynesiensis*, vector of lymphatic filariasis and secondary vector of dengue virus. The aim of this study was to analyze the ability of these two Pacific vectors to transmit ZIKV at a regional scale, through the evaluation and comparison of the vector competence of wild *Ae*. *aegypti* and *Ae*. *polynesiensis* populations from different Pacific islands for a ZIKV strain which circulated in this region during the 2013–2017 outbreak.

**Methodology/principal findings:**

Field *Ae*. *aegypti* (three populations) and *Ae*. *polynesiensis* (two populations) from the Pacific region were collected for this study. Female mosquitoes were orally exposed to ZIKV (10^7^ TCID50/mL) isolated in the region in 2014. At 6, 9, 14 and 21 days post-infection, mosquito bodies (thorax and abdomen), heads and saliva were analyzed to measure infection, dissemination, transmission rates and transmission efficiency, respectively. According to our results, ZIKV infection rates were heterogeneous between the *Ae*. *aegypti* populations, but the dissemination rates were moderate and more homogenous between these populations. For *Ae*. *polynesiensis*, infection rates were less heterogeneous between the two populations tested. The transmission rate and efficiency results revealed a low vector competence for ZIKV of the different *Aedes* vector populations under study.

**Conclusion/significance:**

Our results indicated a low ZIKV transmission by *Ae*. *aegypti* and *Ae*. *polynesiensis* tested from the Pacific region. These results were unexpected and suggest the importance of other factors especially the vector density, the mosquito lifespan or the large immunologically naive fraction of the population that may have contributed to the rapid spread of the ZIKV in the Pacific region during the 2013–2017 outbreak.

## Author summary

Zika virus (ZIKV) was isolated for the first time in Uganda in 1947. The virus was sporadically detected in Africa and Asia during few decades. The first human outbreak was declared in Yap Island in Micronesia in 2007. The virus reemerged in 2013 in French Polynesia and circulated throughout the Pacific region and in Americas between 2013 and 2017. ZIKV is transmitted to human by the bite of an infected mosquito. *Aedes* mosquitoes were particularly incriminated in the ZIKV outbreak. In this study, we showed for the first time the results of vector competence study of wild *Aedes aegypti* and *Aedes polynesiensis* from different islands of the Pacific region. Our results demonstrated an unexpected homogenous and low ZIKV vector competence for the different populations of these two vectors. These findings seemed to indicate that other factors have contributed to the rapid spread of ZIKV in the Pacific region.

## Introduction

Zika fever is an emerging vector borne disease caused by a single stranded RNA virus, Zika virus (ZIKV) belonging to the genus *Flavivirus* [1]. ZIKV is transmitted to humans by the bite of infected vectors mainly *Aedes* mosquitoes [2–4]. Since its first isolation in Uganda in 1947, ZIKV has been detected occasionally in Africa and Asia [5]. The first human outbreak was declared in Yap state in Federated States of Micronesia in 2007 [4]. ZIKV reemerged in the Pacific region in 2013 in French Polynesia and then spread across the region and to the Americas between 2014 and 2016 [5–9]. This recent outbreak was associated with neurological disorders as Guillain-Barré syndrome [10–12], myasthenia gravis [13] and microcephaly [14, 15] were reported in the Pacific region and in South and Central America including the Caribbean (http://www.paho.org). Phylogenetic studies indicated that ZIKV is divided into two lineages: African and Asian with less than 12% of genetic divergence [16, 17]. The 2013–2016 outbreak was due to the emergence of ZIKV belonging to Asian lineage, and genetic analysis revealed divergence between the Pacific and the American clades [18, 19].

During the 2013 French Polynesian ZIKV outbreak, more than 8,700 suspected cases and 30,000 medical consultations were reported by the sentinel surveillance network [10]. A recent seroprevalence study estimated that more than half of the population was infected by ZIKV during the outbreak in this territory [20]. From French Polynesia, the disease spread to New Caledonia in 2013 [7, 21] affecting the whole territory. Based on the number and proportion of confirmed cases and the proportion of ZIKV infections among arboviral syndromes recorded in the population, New Caledonia Health Authorities estimated the number of cases at about 11,000 [11]. From 2014 to 2017, ZIKV was detected in the Cook Islands, Vanuatu, Fiji, Samoa, Salomon Islands, Tonga and American Samoan (http://www.spc.int/phd/epidemics/) [14, 22].

In the Pacific landscape, different mosquito species are present and some of them are known as potential arbovirus vectors, especially mosquitoes from *Aedes* genus. *Ae*. *aegypti* is present in most Pacific islands with few exceptions [23]. The presence of *Ae*. *albopictus* is confirmed in 5 out of 17 countries and territories of this region and its expansion through the Western Pacific islands is observed right up to Fiji and Tonga islands [24, 25]. *Ae*. *polynesiensis* distribution is reported in the Eastern part of the Pacific, from French Polynesia to Fiji [22]. In addition, the presence of other vectors is recorded in some local parts of the Pacific region as *Aedes hensilli*, *Aedes scutellaris* and other *Aedes* species belonging to the scutellaris group ([26–28].

Previous studies evaluated the vector competence of *Aedes* vectors for ZIKV belonging to both lineages. Mosquitoes from Africa [29], Asia [30, 31] and America [32, 33] were infected by ZIKV strains belonging to the African lineage and showed various results. The vector competence for ZIKV belonging to the Asian lineage was also evaluated with vectors from America [2, 34] Australia [35] and Europe [36]. However, the ability of the vectors from the Pacific to transmit ZIKV has been poorly studied. *Ae*. *hensilli* from Yap island, appeared to be able to disseminate African ZIKV strain but the transmission has not been evaluated. However this mosquito was the most collected species in Yap island, Federated States of Micronesia, during the ZIKV outbreak in 2007 (41%) supporting its possible role as a vector during the outbreak [27]. Laboratory strains of *Ae*. *aegypti* and *Ae*. *polynesiensis* from French Polynesia were shown to be able to get infected and disseminate an Asian/Pacific ZIKV strain isolated in French Polynesia in 2013. Furthermore, infectious ZIKV particles have been found in saliva of *Ae*. *aegypti* from French Polynesia as soon as 6 days post-infection [37].

The Pacific landscape promotes specific environmental conditions that have influenced the genetic of the main arboviruses vector *Ae*. *aegypti* [22, 38]. The arbovirus transmission depends on the specific combination of mosquito and virus genotypes [39–41]. At a regional scale, this specific interaction has already been studied for dengue virus and highlighted significant transmission differences between New Caledonian and Polynesian *Ae*. *aegypti* [42]. To our knowledge, no studies have compared the vector competence of several field *Aedes* species from the Pacific region with an Asian/Pacific ZIKV strain that circulated in this region. As the Pacific was at the origin of the ZIKV pandemic, there is a real interest in investigating the interactions between arbovirus and Pacific vectors at a regional scale.

For this purpose, we collected three field populations of *Ae*. *aegypti* in New Caledonia (West Pacific), Samoa (Center) and French Polynesia (East) in order to evaluate and compare the vector competence of several populations of the main Pacific vector. Additionally, we collected two field populations of *Ae*. *polynesiensis* in Wallis (from the Territory of Wallis and Futuna Islands) (Center) and Tahiti (French Polynesia) (East) to evaluate the vector competence of this potential regional vector and to compare the results obtained for these two *Aedes* vectors. We performed these vector competence studies with the ZIKV Pacific strain which circulated in New Caledonia in 2014 and belongs to the Asian/Pacific clade.

## Materials and methods

### Mosquito collections

**Fig 1:**
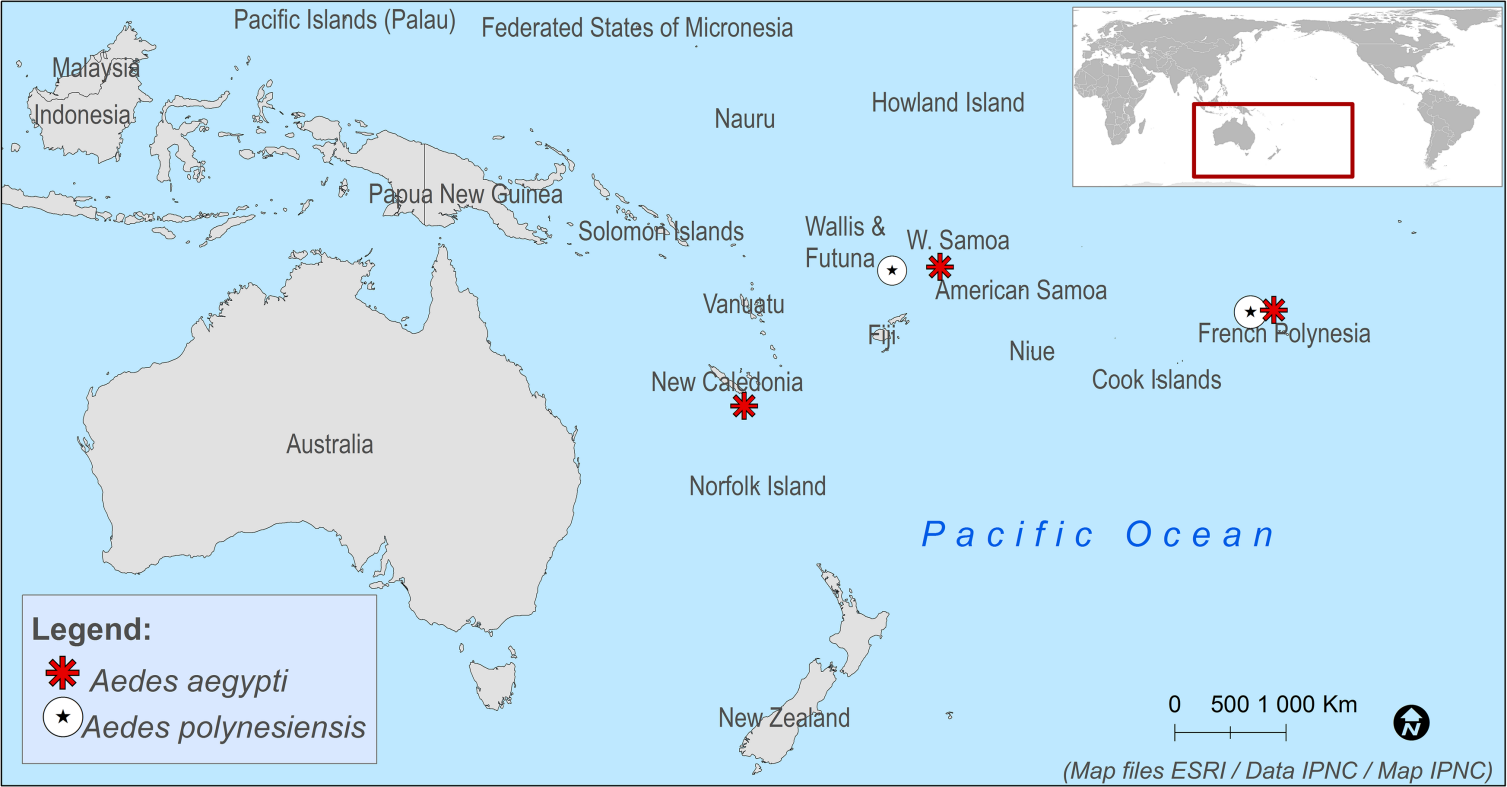
Pacific map locating *Ae*. *aegypti* and *Ae*. *polynesiensis* sampling sites. *Ae*. *aegypti* sampling sites are represented by the red stars and *Ae*. *polynesiensis* sampling sites by the dark star in a white dot. This map was generated using map files provided by ESRI in its ArcMap package.

Mosquitoes were sampled in five sites in the Pacific region in 2016 with permission of the residents and they were shipped to the Laboratory of Arboviruses and Insect Vectors (AIV) at the Institut Pasteur, Paris (Table 1 and Fig 1). The adults were maintained at 28°C and 80% of humidity with a 16:8h light:dark cycle and fed with a 10% sucrose solution *ad libitum*. Females were blood-fed several times to obtain the F1-F3 generation of mosquitoes used for infection assays.

**Table 1:** *Aedes aegypti* and *Aedes polynesiensis* populations sampled in the Pacific region, 2016. When available, data regarding the number of individuals sampled are provided in brackets.

Sample name	Country	Species	Sampled stage	Generation used in laboratory experiments
Aae-French Polynesia	French Polynesia	*Ae*. *aegypti*	Larvae / Pupae (<300)	F_1_
Aae-New Caledonia	New Caledonia	*Ae*. *aegypti*	Larvae / Pupae (<200)	F_2_
Aae-Samoa	Samoa	*Ae*. *aegypti*	Egg	F_3_
Apo-French Polynesia	French Polynesia	*Ae*. *polynesiensis*	Larvae / Pupae (<300)	F_1_
Apo-Wallis	Wallis and Futuna	*Ae*. *polynesiensis*	Adult (160–180 females)	F_3_

### Viral strain

The viral strain used in this study was a ZIKV isolated from a patient’ serum in New Caledonia in April 2014 (NC-2014-5132) (GenBank SRR5309451) (Collection of the Institut Pasteur de Nouvelle-Calédonie). Viral stocks were prepared after five passages on African green monkey kidney cells (Vero E6, ATCC cell lines) maintained in DMEM medium (Gibco) supplemented with 10% fetal bovine serum (FBS) (Gibco). Supernatant were collected after 7 days of incubation at 37°C. The titration of the viral stock was performed by serial 10-fold dilutions on Vero cells and expressed in TCID_50_/mL.

### Mosquito oral infections

For each mosquito population, 4–5 boxes of 60 five to seven day-old females, not previously blood-fed, were starved for 24 hours before infection. They were allowed to take an infectious blood meal, through a capsule (Hemotek system) covered by a pig intestine (obtained from a commercial purchased pig intestine) as membrane and containing 2 ml of washed rabbit erythrocytes obtained directly from a rabbit (New Zeland white Rabbit, Charles River) and 1 ml of viral suspension supplemented with adenosine triphosphate at 5 mM. The ZIKV concentration in the blood meal was 10^7^ TCID_50_/mL. After the blood meal, fully engorged females were transferred into new containers and maintained at 28°C and 80% of humidity under a 12:12h light-dark cycle with free access to a 10% sucrose solution.

### Infection, dissemination and transmission analysis

For each population tested, more than 20 females were analyzed at 6, 9, 14, 21 days post-infection (dpi) and scored infected or non-infected. For each mosquito, abdomen and thorax were analyzed to determine the infection, the head was used for the dissemination and the saliva was tested for the transmission. For all *Aedes* population tested, transmission efficiencies were obtained by the number of infected saliva divided by the total number of mosquitoes tested for each population at each dpi as previously described [42, 43]. For each saliva collection, females were cold anesthetized before removing their legs and wings. The proboscis was inserted into a filter tip ART (Molecular BioProducts) containing 5 μl of FBS for salivation during 20 minutes. The body and the head were individually ground in 250 μL of DMEM medium supplemented with 2% FBS. Lysis was carried out during 30 sec at 6,000 rpm and the samples were centrifuged at 10,000g during 10 min at 4°C. The supernatants were stored at -80°C before analysis. For the viral detection, ground samples serially diluted were inoculated onto Vero E6 cells in 96-well plates, incubated at 37°C during 7 days and stained with a solution of crystal violet (0.2% in 10% formaldehyde and 20% ethanol). Presence of viral particles was determined by the presence of cytopathic effect (CPE). The saliva samples were stored at -80°C. For detection and titration of ZIKV, saliva samples were inoculated onto Vero E6 cells in 6-wells plates under an agarose overlay and incubated at 37°C during 7 days. Presence of infectious particles was assessed by the detection of plaque and titers were expressed as pfu (plaque-forming unit)/saliva.

### Statistical analysis

All rates were statistically compared using Fisher’s exact test (R v. 3.3.1) [44], considering *p*-values > 0.05 non-significant.

### Ethics statement

This study follows the New Caledonia Animal Ethics Guidelines.

## Results

### Different populations of *Aedes aegypti* from the Pacific show similar ZIKV transmission profiles

**Fig 2:**
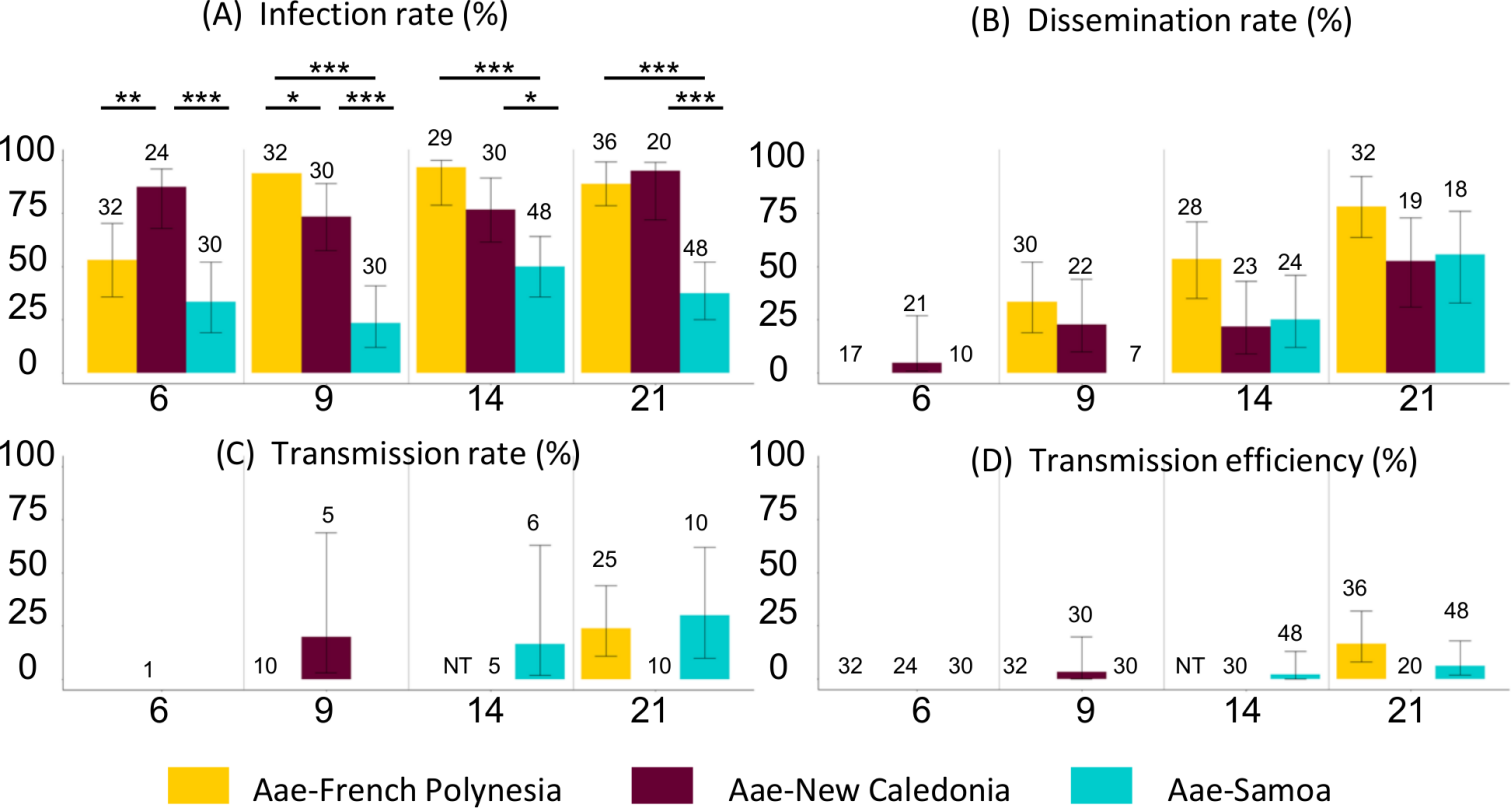
*Aedes aegypti* from the Pacific region infected with ZIKV. **(A) Infection rate, (B) dissemination rate, (C) transmission rate and (D) transmission efficiency at 6, 9, 14 and 21 days post-infection (dpi).** Error bars represent 95% confidence intervals. Numbers of mosquitoes tested are indicated above each bar plot. Significant differences are indicated by asterisks (*p < 0.05; **p < 0.01; *** *p* < 0.001). NT indicates that females were not tested for this analysis point.

The ZIKV infection rates of the French Polynesian and the New Caledonian populations of *Ae*. *aegypti* appeared to be moderate to high (> 53%) (S1 Table and Fig 2A). Significant differences were found between these two mosquito populations at 6 dpi (French Polynesia 53% and New Caledonia 87%, p = 0.009) and 9 dpi (French Polynesia 93% and New Caledonia 73%, p = 0.04). The ZIKV infection rate was significantly lower for the Samoan *Ae*. *aegypti* during all the course of infection (with a minimal value of 23% at 9 dpi and a maximal value of 50% at 14 dpi).

The dissemination rates were comparable between the three *Ae*. *aegypti* populations, although the population of Samoa started to be infected only from 14 dpi (S1 Table and Fig 2B). The dissemination rates of *Ae*. *aegypti* increased during all the course of infection and reached moderate to high levels at 21 dpi (between 53% and 78%).

Infectious viral particles were found in the saliva from 9 dpi for *Ae*. *aegypti* from New Caledonia. The transmission rates for the three populations tested were low and homogenous (< 30% at 21 dpi) (S1 Table and Fig 2C). The transmission efficiencies of these populations were low, from 3% for the Caledonian population (at 9 dpi) to 6% for the Samoan population and 17% for the French Polynesian one (at 21 dpi), and no significant difference was found (S1 Table and Fig 2D). However, the extrinsic incubation period seemed shorter for the Caledonian mosquitoes (9 dpi) compared to the two other populations (> 9 dpi).

### Low and homogenous transmission of ZIKV for *Aedes polynesiensis* from the Pacific region

**Fig 3:**
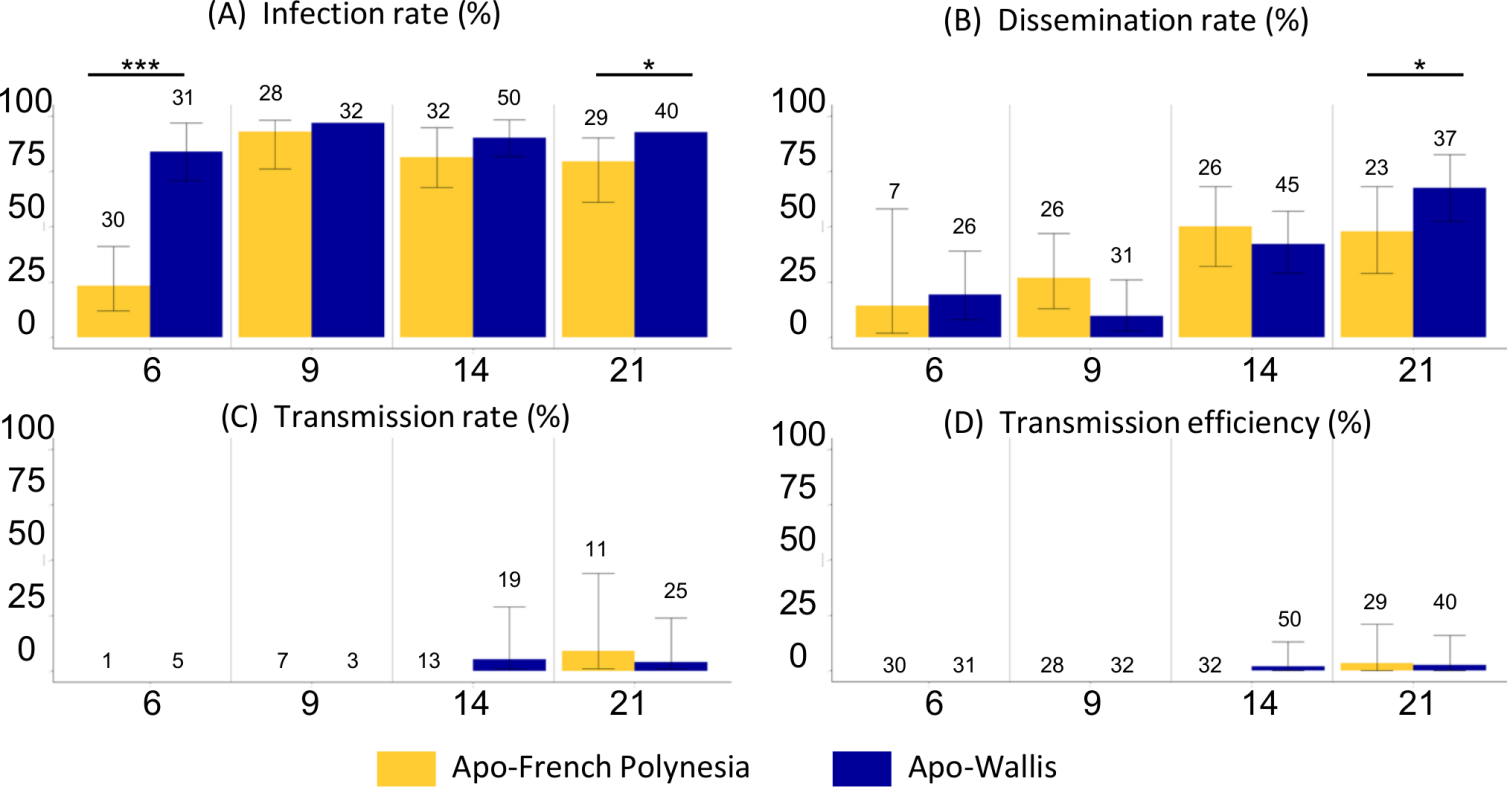
*Aedes polynesiensis* from the Pacific region infected with ZIKV. **(A) Infection rate, (B) dissemination rate, (C) transmission rate and (D) transmission efficiency at 6, 9, 14 and 21 days post-infection (dpi).** Error bars represent 95% confidence intervals. Numbers of mosquitoes tested are indicated above each bar plot. Significant differences are indicated by asterisks (*p < 0.05; **p < 0.01; *** *p* < 0.001).

The ZIKV infection rates appeared to be high and homogenous from 9 dpi for both the Wallisian and French Polynesian populations of *Ae*. *polynesiensis* (S2 Table and Fig 3A). At 6 dpi, ZIKV infection rate of the Wallisian vector was higher than that of the Polynesian population (84% vs 23%, p < 0.001).

The ZIKV dissemination rates increased gradually in both vector populations (S2 Table and Fig 3) with no significant difference, except at 21 dpi (p = 0.0135). Although the infection rates were high, dissemination rates were moderate, even at 21 dpi.

Infectious particles in the saliva were found from 14 dpi. Transmission rates were low for the two *Ae*. *polynesiensis* populations (< 10% at 21 dpi) (S2 Table and Fig 3C). As for *Ae*. *aegypti* populations from the Pacific region, the ZIKV transmission efficiency values for *Ae*. *polynesiensis* were low, 2% and 3% (from 14 dpi) for the studied populations (S2 Table and Fig 3D).

Finally, when comparing both results obtained for each population of *Ae*. *aegypti* and *Ae*. *polynesiensis*, we observed significant differences between the population of *Ae*. *aegypti* from Samoa and both *Ae*. *polynesiensis* populations after 9 dpi for ZIKV infection. Concerning the ZIKV dissemination, few significant differences were observed for the last analysis point (21 dpi) between *Ae*. *aegypti* from New Caledonia and *Ae*. *polynesiensis* from Wallis (p = 0.044) and between *Ae*. *aegypti* and *Ae*. *polynesiensis* from French Polynesia (p = 0.041). However, no significant difference was found between the two mosquito species for ZIKV transmission.

## Discussion

Recently, the tropical islands, especially of the Pacific region, have been incriminated as new hubs for arboviruses emergence [45]. Indeed the ZIKV outbreak in 2013 emerged in French Polynesia and subsequently spread through the Pacific region and to Latin America [5, 6, 19]. In this context, the evaluation of *Aedes* vector competence for the arboviruses that circulated in this region appeared to be pivotal. Recent studies estimated the vector competence of the Pacific vectors for different arboviruses at the island scale for dengue virus in New Caledonia and French Polynesia [42], for chikungunya virus in New Caledonia, French Polynesia, and Yap island [27, 46, 47] and for ZIKV in Yap island and French Polynesia [27, 37].

The quick spread of ZIKV in the Pacific region subsequently to the French Polynesian outbreak highlighted the necessity to evaluate the ZIKV vector competence at the regional scale. This study is the first to describe and compare the ZIKV vector competence of *Ae*. *aegypti* and *Ae*. *polynesiensis* populations from different Pacific islands for a ZIKV strain which circulated in the region during the 2013–2015 outbreaks.

Our results confirmed that *Ae*. *aegypti* populations from New Caledonia, Samoa and French Polynesia can transmit ZIKV but with low transmission efficiencies (< 17% until 21 dpi). In addition, even if the infection rates seemed to indicate a better infection of the New Caledonian and the French Polynesian *Ae*. *aegypti* populations, the transmission efficiency was similar for all populations tested according to our tests. Similar results were obtained in the present study for *Ae*. *polynesiensis* with infectious viral particles found in the two populations tested, thus demonstrating the potential role of this mosquito species in transmitting ZIKV (< 3% until 21 dpi). For *Ae*. *aegypti*, the infection rates seemed to indicate heterogeneous profiles, this profile was more homogeneous for *Ae*. *polynesiensis*. The Samoan population of *Ae*. *aegypti* and the French Polynesian population of *Ae*. *polynesiensis* seemed to be less susceptible to the infection than the populations from the other study sites. A previous study performed in French Polynesia with *Ae*. *aegypti* showed higher ZIKV transmission rates (3, 8, 36 and 73% at 6, 9, 14 and 21 dpi) [37]. Such discrepancies may be explained by differences in the experimental protocol: the previous competence work was performed using F16 to F18 generation mosquitoes; moreover, the detection of infectious particles in mosquito saliva was performed on C6/36 instead of Vero cells. The fact that previous experiments were conducted using the French Polynesian instead of New Caledonian ZIKV strain may also be another possible explanation to such dissimilarity, but in both studies the vector competency for ZIKV was confirmed. In contrast to DENV that was previously found as highly disseminating in the New Caledonian and French Polynesian *Ae*. *aegypti*, the dissemination rates for ZIKV indicated limited escape from the midgut barrier [42]. Finally, Australian *Ae*. *aegypti* has been recently tested for ZIKV transmission [35]. The results showed high prevalence of virus in the saliva. However, the ZIKV strains used although belonging to the Asian lineage was isolated in Cambodia in 2010 (before ZIKV outbreak) thus not harboring the specific amino acids substitution of the Asian American or Pacific sublineage [18, 19]. Differences in the susceptibilities to ZIKV depending on the mosquito species and population, suggest that the outcome of mosquito infection may vary depending on virus-mosquito pairing described as genotype-by-genotype interaction [48]. Moreover, the efficiency of ZIKV in overcoming the midgut infection and escape barrier may be influenced by several factors, notably the microbiome [49, 50] and innate immunity [51, 52] which could be both related to the breeding site environment [53, 54]. The role of the salivary glands as a barrier seemed to be observed for all the populations tested.

Since the explosive ZIKV outbreaks, the vector competence of several mosquito species and populations for this virus has been tested. Previous results highlighted some heterogeneity in the ZIKV transmission, especially due to the ZIKV lineage [55]. In this study, the aim of the experiments was to test the vector competence of Pacific mosquitoes for the ZIKV which circulated during the outbreak (Asian/Pacific lineage). Our results corroborate previous findings that mosquito vectors, whatever the area they originate from (Pacific, America, Europe), are mostly poorly competent for the Asian/Pacific sublineage of ZIKV [2, 36, 37]. We did not have the opportunity here to test *Ae*. *albopictus* from the Pacific region. Although, *Ae*. *albopictus* could be relevant to Public Health as a potential invasive species in the region, it might not have sustained an outbreak by its own as it is not present in New Caledonia and French Polynesia [22] and as it appears to be a poor ZIKV vector in other region [55]. All together, these observations support that the emergence of ZIKV in the Pacific region and other regions, did not solely rely on the competence of the vectors to transmit this virus. Several other factors, notably the vector density, the mosquito lifespan and non-entomological parameters like the large immunologically naive fractions of the population may have contributed to the unexpected globalization of ZIKV. This work highlights the necessity to characterize the interaction between mosquitoes, arboviruses and humans in the areas recently affected in order to better assess the risk for such arboviruses to emerge and to limit the burden of future outbreaks.

## Supporting information

S1 Table Infection, dissemination, transmission rates and transmission efficiency at 6, 9, 14 and 21 days post-infection (dpi) for *Aedes aegypti* Pacific populations. (DOCX)

S2 Table Infection, dissemination, transmission rates and transmission efficiency at 6, 9, 14 and 21 days post-infection (dpi) for *Aedes polynesiensis* Pacific populations. (DOCX)
