# Supplementary material for: Zika virus outbreak in the Pacific: Vector competence of regional vectors
Source: PLoS Negl Trop Dis. 2018 Jul 17;12(7):e0006637. doi: 10.1371/journal.pntd.0006637 (PMC6063428; doi:10.1371/journal.pntd.0006637)
Supplement: S1 Table — (DOCX) [file pntd.0006637.s001.docx]

|  |  | 6 dpi | 9 dpi | 14 dpi | 21dpi |
| --- | --- | --- | --- | --- | --- |
| % of infection  (Number of infected bodies / number of mosquitoes tested) | Aae-New Caledonia | 88% (21/24) | 73% (22/30) | 77% (23/30) | 95% (19/20) |
|  | Aae-Samoa | 33% (10/30) | 23% (7/30) | 50% (24/48) | 38% (18/48) |
|  | Aae-French Polynesia | 53% (17/32) | 94% (30/32) | 97% (28/29) | 89% (32/36) |
| % of dissemination  (Number of infected heads / number of infected bodies) | Aae-New Caledonia | 5% (1/21) | 23% (5/22) | 22% (5/23) | 53% (10/19) |
|  | Aae-Samoa | 0% (0/10) | 0% (0/7) | 25% (6/24) | 56% (10/18) |
|  | Aae-French Polynesia | 0% (0/17) | 33% (10/30) | 54% (15/28) | 78% (25/32) |
| % of transmission  (Number of infected saliva / number of infected heads) | Aae-New Caledonia | 0% (0/1) | 20% (1/5) | 0% (0/5) | 0% (0/10) |
|  | Aae-Samoa | 0% (0/0) | 0% (0/0) | 17% (1/6) | 30% (3/10) |
|  | Aae-French Polynesia | 0% (0/0) | 0% (0/10) | 0% (0/15) | 24% (6/25) |
| % of efficiency  (Number of infected saliva / number of mosquitoes tested) | Aae-New Caledonia | 0% (0/24) | 3% (1/30) | 0% (0/30) | 0% (0/20) |
|  | Aae-Samoa | 0% (0/30) | 0% (0/30) | 2% (1/48) | 6% (3/48) |
|  | Aae-French Polynesia | 0% (0/32) | 0% (0/32) | 0% (0/29) | 17% (6/36) |

**Table S1*.* Infection, dissemination, transmission rates and transmission efficiency at 6, 9, 14 and 21 days post-infection (dpi) for *Aedes aegypti* Pacific populations.**

NT indicates that females were not tested for this analysis point.
