# Supplementary material for: Zika virus outbreak in the Pacific: Vector competence of regional vectors
Source: PLoS Negl Trop Dis. 2018 Jul 17;12(7):e0006637. doi: 10.1371/journal.pntd.0006637 (PMC6063428; doi:10.1371/journal.pntd.0006637)
Supplement: S2 Table — (DOCX) [file pntd.0006637.s002.docx]

**Table S2*.* Infection, dissemination, transmission rates and transmission efficiency at 6, 9, 14 and 21 days post-infection (dpi) for *Aedes polynesiensis* Pacific populations.**

|  |  | 6 dpi | 9 dpi | 14 dpi | 21dpi |
| --- | --- | --- | --- | --- | --- |
| % of infection  (Number of infected bodies / number of mosquitoes tested) | Apo-French Polynesia | 23% (7/30) | 93% (26/28) | 81% (26/32) | 79% (23/29) |
|  | Apo-Wallis | 84% (26/31) | 97% (31/32) | 90% (45/50) | 93% (37/40) |
| % of dissemination  (Number of infected heads / number of infected bodies) | Apo-French Polynesia | 14% (1/7) | 27% (7/26) | 50% (13/26) | 49% (11/23) |
|  | Apo-Wallis | 19% (5/26) | 10% (3/31) | 42% (19/45) | 68% (25/37) |
| % of transmission  (Number of infected saliva / number of infected heads) | Apo-French Polynesia | 0% (0/1) | 0% (0/7) | 0% (0/13) | 9% (1/11) |
|  | Apo-Wallis | 0% (0/5) | 0% (0/3) | 5% (1/19) | 4% (1/25) |
| % of efficiency  (Number of infected saliva / number of mosquitoes tested) | Apo-French Polynesia | 0% (0/30) | 0% (0/28) | 0% (0/32) | 3% (1/29) |
|  | Apo-Wallis | 0% (0/31) | 0% (0/32) | 2% (1/50) | 3% (1/40) |
